# Silicea

**Published:** 1898-06

**Authors:** 


### HOMŒOPATHIC PHYSICIAN,

A MONTHLY JOURNAL OF

HOMŒOPATHIC MATERIA MEDICA AND CLINICAL MEDICINE.

"If our school ever gives up the strict inductive method of Hahnemann, we are lost, and deserve only to be mentioned as a caricature in the history of medicine."—CONSTANTINE HERING.

Vol. XVIII. JUNE, 1898. No. 6.

### EDITORIAL.

SILICEA has short flushes of heat in the face, happening several times a day. Fluoric-acid, Lachesis, and Phosphorus have burning heat of the face. Fluoric-acid, with the heat is desire to wash the face with cold water. Lachesis, the heat in the face is attended with great sensitiveness of the neck to touch. Phosphorus, the heat begins in the hands and spreads all over the body, including the face.

Silicea has debilitating perspiration in the morning hours of the night. Calcarea-carb. has perspiration before midnight as before stated.

Silicea is generally worse in the morning.

Kali-carbonicum has perspiration after midnight.

Silicea has painless swelling of the glands. Calcarea has swelling with heat, and Belladonna swelling with heat and redness.

Silicea has rose-colored blotches on the skin. Natrum-carb. has red, hard blotches.

Silicea has ulcers relieved by heat and made worse by cold.

Fluoric-acid has ulcers relieved by cold.

Arsenic, the ulcers are relieved by heat.

Iodine, ulcers worse from heat.

Silicea, scars left from old ulcerations suddenly become painful.

Silcea has aggravation from lifting the diseased limb.

Silicea has aggravation from washing.

Fluoric-acid has amelioration from cold washing.

Here are some indications derived from washing. Those who practice Homœopathy will be likely to find them useful. Those who do not practice Homœopathy need not read them, as they will hardly make use of them, and it is not well to crowd the mind with information that is not useful.

Sulphur, aversion to washing. Also Antimonium-crudum according to Dr. Guernsey, and Ammonium-carb.

Ammonium-carb. has reappearance of old symptoms from washing, such as nose-bleed, blue hands, and swollen face.

Aloes, patient can't take a cold bath because it is too stimulating to the genitals.

Hepar has utter aversion to bathing; the child screams and fights if it be attempted, on account of eruption upon the head.

Kali-carbonicum, washing in cold water causes red spots on the face.

Kali-carb., washing the face causes nose-bleed.

Phytolacca, blotches on face worse from washing.

Sarsaparilla, eruptions worse from washing.

Hydrastis, washing aggravates eruption on forehead.

Lobelia, cold washing increases or causes pains and difficulty of breathing.

Lycopodium aggravates from moistening the diseased part.

Antimonium-crudum child cries, when washed in cold water and makes but little objection to washing in warm water.

Natrum-muriaticum has desire to wash in cold water.

Borax, washing chest with cold water relieves chest symptoms.

Æsculus, after washing hands and face swell up enormously and become red. Red spots on face after washing. Rubbing the face after washing brings red spots.

Apis, desire to wash the face in cold water. The face is better from washing or moistening.

Nux-moschata, amelioration of colic by application of hot, wet cloths, and aggravation from cold, wet cloths.

Aconite and Fluoric-acid, better from washing the diseased part.

Fluoric-acid, desire to have the suffering part bathed.

Thuja, aggravation from cold, wet applications.

Thuja, amelioration from warm, wet applications.

Asarum-europeum, eyes relieved by washing in cold water.

Sulphur, eyes worse from washing, or bathing.

Thuja, face feels sore and raw when washing it.

Antimonium-crudum, tendency to take cold in the head from washing. The head is sensitive to cold bathing.

Cantharis, headache from washing.

Aconite, cold bathing suppresses the menses.

Aurum-m., many symptoms disappear after washing.

Muriatic-acid, washing with warm water ameliorates the hemorrhoids. Cold water aggravates the hemorrhoids.

Silicea has amelioration from letting the diseased limb hang down.

Pulsatilla has aggravation from letting diseased limb hang down.

Indium-metallicum has pulsation in foot from letting it hang down.

Natrum-carbonicum has aggravation from letting diseased limb hang down.

Conium has amelioration from letting diseased limb hang down, in cases of periostitis with throbbing and burning pain.

